# Novel Road Traffic Management Strategy for Rapid Clarification of the Emergency Vehicle Route Based on V2V Communications

**DOI:** 10.3390/s21155120

**Published:** 2021-07-28

**Authors:** Radwa Ahmed Osman, Amira I. Zaki, Ahmed Kadry Abdelsalam

**Affiliations:** 1 Basic and Applied Science Department, College of Engineering and Technology, Arab Academy for Science and Technology (AAST), Alexandria 1029, Egypt; 2 Electronics and Communications Department, College of Engineering and Technology, Arab Academy for Science and Technology (AAST), Alexandria 1029, Egypt; amzak10@aast.edu; 3 Electrical and Control Engineering Department, College of Engineering and Technology, Arab Academy for Science and Technology (AAST), Alexandria 1029, Egypt; ahmed.kadry@aast.edu

**Keywords:** emergency-vehicle, vehicular communication, road safety, vehicle-to-vehicle (V2V), vehicle-to-infrastructure (V2I), reliability, efficiency, travel time, packet delivery ratio, average end to-end delay

## Abstract

Vehicle-to-vehicle communication is a promising paradigm that enables all vehicles in the traffic road to communicate with each other to enhance traffic performance and increase road safety. Through vehicle-to-vehicle (V2V) communication, vehicles can understand the traffic conditions based on the information sent among vehicles on the road. Due to the potential delay caused by traffic jams, emergency vehicles may not be able to reach their destination in the required time, leading to severe losses. The case is more severe especially in developing countries where no emergency-vehicle-dedicated lanes are allocated. In this study, a new emergency vehicle route-clarifying strategy is proposed. The new clarifying strategy is based on vehicular traffic management in different interference medium scenarios. The proposed model aims, through V2V communication, to find the nearest vehicle with which to communicate. This vehicle plays an important role in reducing the travel time: as the emergency message is received, this vehicle will immediately communicate with all the neighboring vehicles on the road. Based on V2V communications, all the vehicles in the road will clear from the lane in the road for the emergency vehicle can safely reach its destination with the minimum possible travel time. The maximum distance between the emergency vehicle and the nearest vehicle was determined under different channel conditions. The proposed strategy applied an optimization technique to find the varied road traffic parameters. The proposed traffic management strategy was evaluated and examined through different assumptions and several simulation scenarios. The obtained results validated the effectiveness and the accuracy of the proposed model, and also indicated significant improvement in the network’s performance in terms of packet delivery ratio (PDR) and average end-to-end delay (E2E).

## 1. Introduction

The last few decades have witnessed a huge rise in the population living in the same metropolitan areas, which has increased the road traffic density and, consequently, has increased the number of accidents year by year. Most governments and the automotive industry invest many resources into increasing road safety and traffic efficiency, and also reducing the harmful effects of the transportation system on the environment. For this reason, vehicle communication and vehicle ad-hoc networks (VANETs) aim to improve road safety, driving comfort, and traffic efficiency [1]. One of the everincreasing problems worldwide is traffic congestion. Congestion increases fuel consumption and travel time due to traffic jams, increases air pollution, reduces the efficiency of the transportation infrastructure, and affects people’s health. Increasing vehicle numbers worldwide have significantly increased the number of accidents and decreased the safety of vehicles and pedestrians [2]. Additionally, they increase the network’s overhead and interference with other vehicles. Vehicle-to-Everything (V2X) is considered as one of the promising techniques for intelligent transportation system (ITS) that would enable vehicles, infrastructure, and people to exchange information. Moreover, V2X mainly includes vehicle-to-vehicle (V2V), vehicle-to-infrastructure (V2I), vehicle-to-device (V2D), vehicle-to-pedestrian (V2P), and vehicle-to-network (V2N) communication. All these applications require efficient, reliable, scalable, and timely communication systems [3].

V2V networks have become a very common technique for improving traffic conditions and safe driving by sharing road and traffic information among vehicles in real time. Additionally, when V2V communications are applied, there is no need for roadside units or infrastructure to transfer the information to other vehicles; hence, vehicles can be used as relays to directly send and exchange the information [4]. Furthermore, to increase road safety and improve road traffic, it is important to link the characteristics of V2V communications with the physical mobility characteristics of the vehicular system through developing new frameworks [5]. Moreover, the detection of the road traffic congestion through V2V communication plays an important role in reducing the network’s overhead by distributing congestion information [6].

Dedicated short-range communication (DSRC) is used to support V2I and V2V communications [7,8]. Based on the IEEE 802.11p, the DSRC system adopts the carrier sense multiple access (CSMA) technique to help vehicles to directly communicate with each other without the need to go through the infrastructure [9]. Additionally, the physical layer of IEEE 802.11p can be implemented to mitigate the effect of imperfect channel state information (CSI) [10]. Furthermore, DSRC can be considered as a very attractive feature through developing a new approach that leads to a cost-effective solution for urban traffic control [11]. Since the performance of DSRC is directly related to human and vehicle safety, its elaborated performance was evaluated in real-world scenarios [12].

During a traffic jam, if an emergency vehicle becomes stuck and its arrival at the accident location is delayed, it may cause victims and loss of property. Thus, this work proposes a new vehicular traffic management system for emergency vehicles such as ambulance vehicles, fire engines, and police cars, based on V2V communication, to solve the problem of travel delays due to traffic jams. The contributions of this article are summarized as follows.

- The proposed approach allows the emergency vehicles to communicate with the nearest vehicle in the crowded area to effectively send an emergency message to other vehicles, containing the information that there is an emergency vehicle on its way. Upon receiving this information, all the vehicles on the road will change their paths and clear the path for the oncoming emergency vehicle.
- The proposed approach developed an efficient method to enhance transmission efficiency and system reliability, and reduce the travel time of the emergency vehicles to drive safely to their destination, which saves people’s lives. This can be achieved by finding the nearest vehicles to communicate with and efficiently relay messages to the other vehicles on the road.
- An optimization problem to ascertain that the communication among all the vehicles is reliable, and to achieve the required system QoS was formulated.
- The proposed technique was evaluated in terms of packet delivery ratio, average end-to-end delay, and travel time under different conditions, such as differing transmission power, channel conditions (path loss exponent), and interference due to the other transmission sources. The interference can be, for example, from vehicles or devices, vehicle mobility, vehicle density, and vehicle speed. These findings can optimize the system performance for the whole network in a vibrant environment.

The proposed paper is organized into five sections. Following the introduction, Section 2 discusses the relevance of this research to other work. The system model, optimization problem formulation, and the proposed scenario for reducing the travel time are presented in Section 3. Simulation results and discussions are provided in Section 4. Finally, the paper is concluded in Section 5.

## 2. Literature Review

Different algorithms and protocols have been proposed in literature regarding traffic congestion. A recent evolved criterion called traffic processability, which implies a balance between the traffic demand and traffic capacity, has been presented to mitigate road traffic congestion [13]. This model was implemented based on utilizing the traffic processability of the neighbors, where different weights are assigned to roads in each agent according to their importance or the real-time traffic conditions. Additionally, to decrease travel delays, a traffic guidance method was proposed and a Nash equilibrium optimization scheme was designed, which succeeded in reducing the travel time during the traffic congestion [14]. Furthermore, to improve mobility in large urban traffic systems, a two-level hierarchical model-based predictive control system was presented to improve the whole network’s performance under different traffic scenarios [15]. Moreover, to predict the probability of traffic congestion, discrete-time Markov chain and online traffic monitoring was used to optimize the vehicle routes as presented [16]. Additionally, the traffic signal control strategy proposed in [17] aimed to minimize the vehicle travel time and increase road safety based on implementing a macroscopic model for pedestrian–vehicle mixed-flow networks. Furthermore, machine learning was considered as one of the promising techniques to control traffic congestion and, at the same time, to attempt to guarantee the system’s quality-to-service requirements. Machine learning requires large datasets to be used in the training steps which is solved in [18].

Improving the efficiency of vehicular communications through a traffic jam is another important issue that should be studied and addressed, as traffic jamming causes channel overhead, and congestion reduces the system’s reliability and increases the average end-to-end delay. A dynamic link reliability method, called a link reliability-based adaptive routing algorithm (LRAR), was presented to enhance the transmission efficiency by increasing the packet delivery ratio and reducing the average end-to-end delay [19]. Additionally, to enhance the transmission efficiency of VANETs, a new framework of content delivery was suggested, where each moving vehicle can obtain small-volume content files from either the nearest infrastructure (base station) or roadside unit. Next, a stochastic geometry and point process theory was implemented to establish the profit models for both base stations and roadside units [20]. For improving the quality of service (QoS) across vehicular communications, a model was proposed, based on cooperative communications for a combined vehicle-to-infrastructure (V2I) system in which vehicle-to-vehicle communication was elaborated to increase the system’s reliability and transmission efficiency [21,22].

Furthermore, V2V communication is a promising technique that enhances V2I communication and also helps improve the overall system performance of vehicular communications. Additionally, it helps improve road safety conditions, prevent accidents, and overcome human errors. In addition, through implementing a stochastic model of V2V and V2I communications, the system’s reliability can be enhanced by merging the effects of channel contentions and vehicle mobility [23]. To guarantee the QoS of V2I and V2V communications, a reinforcement learning (RL) framework based on statistical information and a slow fading parameter under different channel conditions succeeded in maximizing the bandwidth of V2I communication and the reliability of V2V links [24].

On the other hand, cooperative communication has been considered as one of the promising techniques for system performance improvement. A new cooperative transmission scheme called cooperative superposed transmission (CST) allows vehicle users to retransmit other users’ V2V packets during their transmission to achieve high system reliability and low latency [25]. Moreover, a cooperative communication scheme with optimized resource utilization has been suggested to increase the system’s throughput through investigating interference management and resource allocation based on a clustering mechanism in the D2D communications underlying VANETs [26]. Furthermore, a cooperative solution for V2X guaranteed both reliability and latency reduction for 5G and V2X, consequently decreasing the transmission collision probability [27]. Additionally, transmission of the information from the source to its destination over multi-hop V2V communication through the best relay vehicle selection was analyzed with the aim of enhancing the system’s performance and the system’s accuracy [28].

This study presents a new traffic management system for traffic congestion based on V2V communication for emergency vehicles. The proposed model aims to allocate the nearest vehicle for the emergency vehicle to communicate with under different conditions such as network capacity and interference. The proposed role of this vehicle is to send emergency vehicle messages to other vehicles in the road with reliable and efficient connection and minimum end-to-end delay. The proposed strategy applies an optimization technique to optimize the maximum distance between any two successive vehicles in the route based on the various road traffic parameters in terms of three aspects: (i) the maximum safe distance between every two successive vehicles, (ii) the required maximum limited velocity, and (iii) the maximum possible number of vehicles in each lane. Thus, based on the received message; all vehicles on the road will follow another path and clear the way for the emergency vehicle. This will enhance the overall vehicular system’s performance and, consequently, decrease the travel time needed for the emergency vehicle to arrive safely at its destination. Different scenarios were investigated to assess and evaluate the overall performance of the proposed approach in terms of packet delivery ratio and average end-to-end delay. The proposed model shows how to achieve the optimized required vehicular communication performance through finding the nearest vehicle to the emergency vehicle for relaying the message to other vehicles. Consequently, the overall communication system’s efficiency and reliability are enhanced. Additionally, the proposed approach guarantees adequate clearing of the path for the emergency vehicles to reach their destinations with minimum travel time. This proposed model is a critical solution for saving people’s lives and property, especially in developing countries, where no dedicated lanes for emergency vehicles are allocated.

## 3. Proposed Model and Problem Formulation

### 3.1. System Model

**Figure 1:**
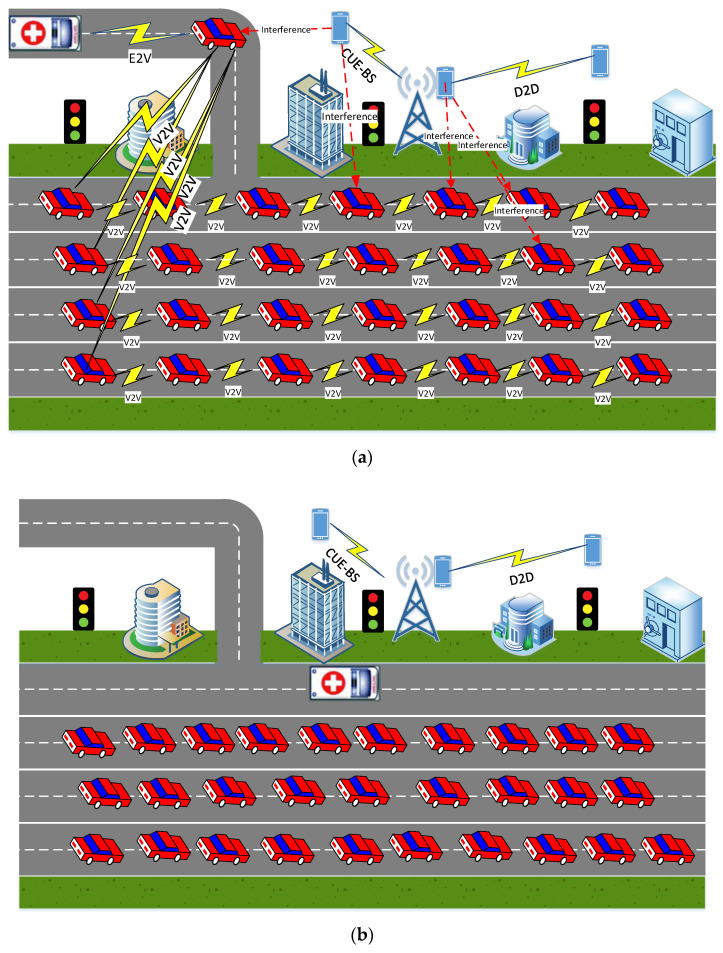
System model for emergency vehicle communications (**a**) before receiving the emergency message and (**b**) after receiving the emergency message.

Traffic jams and interference may affect people lives and the reliability of communication. Traffic jams can prohibit emergency vehicles from reaching their destination in minimum travel time. On the other hand, the interference that occurs due to other devices sharing the same spectrum as the sender may lead to reception of inappropriate information. Thus, the objective of the proposed model, as shown in Figure 1, is to reduce the travel time of the emergency vehicles in the traffic jam. Additionally, the proposed model aimed to enhance the V2V connectivity, which could be affected by the interference caused by other vehicles or devices that share the same spectrum. Therefore, the model is capable of evaluating the nearest vehicle to the emergency vehicle with which it can communicate under different conditions such as different network capacity and interference, as shown in Figure 1a. The distance between the nearest vehicle and the emergency vehicle should be affected by different parameters such as channel conditions, interference, path loss, and transmission power. Furthermore, depending on the nearest vehicle’s position to the emergency vehicle, it should be able to relay the emergency message to the vehicles in the traffic jam. After receiving the emergency message, the vehicles in the road will then follow another path and clear a specific path for the emergency vehicle to drive safely and promptly to its destination, as shown in Figure 1b. This should reduce the travel time, which would save peoples’ lives and property. The proposed model was evaluated in terms of packet delivery ratio and average end-to-end-delay via the combined use of V2V communications, vehicle mobility, vehicle density, vehicle speed, and different interference levels.

A vehicular network was considered that consisted of a road with 4 lanes; V2X and D2D communications were considered in the proposed model. The width of a lane ranged from 3.0 to 3.5 m for any road [29]; this width is typically small compared with the transmission communication range. Due to traffic jams, vehicles were present all along the road and the distance between every two vehicles followed the 2 s rule, where the driver should drive at least 2 s behind the vehicle in front during ideal conditions based on the rules of different countries [30].

### 3.2. Problem Formulation

The goal of the proposed model was to find the nearest vehicle to the emergency vehicle (E2V) with which the emergency vehicle can communicate under different conditions such as different network capacity and interference. Based on the vehicle’s position, it should relay the emergency message to the other vehicles in the traffic road with maximum required quality-of-service (*QoS*) and maximum interference transmission power to effectively send the information with the highest packet delivery ratio (PDR) and minimal packet average end-to-end delay (E2E), i.e.,

Max Σ *d_VnVn+1_*  *d_VnVn+1_*: = *f*(*d_VnVn+1_*, *P_v_*, ***𝛌***, *μ*) s.t. C1: {*p_out_* ≤ *p_outmax_*} C2: {*P_v_* ≤ *P_vmax_*} (1)

In the formulated optimization problem, C1 is the constraint that the system outage probability (*p_out_*) should be less than or equal to the maximum outage probability (*p_outmax_*) to satisfy the required *QoS*. C2 indicates the constraint that the maximum transmission power of any transmission vehicle on the road (*P_v_*) must be lower than the maximum vehicle transmission power (*P_v__max_*). *d_VnVn+_*_1_ is the distance between any two successive vehicles on the road. *λ* and *μ* are the non-negative Lagrangian multipliers for C1 and C2, respectively.

The required *QoS* can be expressed as [31]:

*QoS* = 1 − *p_out_* (2)

Additionally, *d_VnVn+_*_1_ can be derived from the equation given in [32]:

(3) $$d_{VnVn+1}={\left(\frac{-SINR_{th}\left(P_{D}{\left|h_{DVn+1}\right|}^{2}\;PL_{DVn+1}+P_{C}{\left|h_{CVn+1}\right|}^{2}\;PL_{CVn+1}+P_{vi}{\left|h_{VVn+1}\right|}^{2}\;PL_{VVn+1}+N\right)}{P_{v}{\left|h_{VnVn+1}\right|}^{2}\;PL_{O}\;ln\left(1-p_{out}\right)}\right)}^{-\frac{1}{\alpha }}$$

where *p_out_* is the system outage probability. The system outage probability is defined as the probability that the signal-to-interference-plus-noise-ratio (*SINR*) at the receiver is less than the required threshold (*SINR_th_*) which allows error-free decoding. Hence, *p_out_* can be given by [33,34]:

(4) $$\;p_{out}=p\left(SINR\leq SINR_{th}\right)=1-e^{-\left(\frac{SINR_{th}\;\left(N+I\right)}{P_{s\;}{\left|h\right|}^{2}\;\gamma }\right)}$$

where *γ* and *I* are the path loss between the source and destination and the interference which occurs due to the other sources that share the same spectrum. *N* is the noise power spectral density. *h* and *P_S_* represent the fading channel coefficient between the source and destination, and the transmission power, respectively.

The proposed model tests system efficiency and reliability under various operating conditions using standard benchmarks such as (i) packet delivery ratio, and (ii) average end-to-end delay.

We assume that the transmission signal between V2V links undergoes Rayleigh fading with additive white Gaussian noise (AWGN) with zero mean, variance *N_O_*, and propagation path loss [35]. The channel fading is considered to be statistically mutually independent for different links. Furthermore, as a wireless standard for vehicular communications, DSRC has been designed to support the automotive system. DSRC technology involves different types of short-range wireless communication channel, which are one-way and two-way. These two types are designed for automotive communication. The 75 MHz spectrum in the 5.9 GHz band for short-range communications has been allocated by the US Federal Communications Commission (FCC) to be used in intelligent transportation systems (ITS) including V2I and V2V links. Furthermore, 30 MHz of the spectrum in the 5.9 GHz band for ITS has been allocated by the European Telecommunications Standards Institute (ETSI). IEEE 802.11p standardizes the physical and MAC layer of DSRC for vehicular communication. Additionally, it aims to provide a communication range up to 1000 m for both V2V and V2I communications; it also supports transmission rates from 3 to 27 Mb/s over a bandwidth of 10 MHz [10].

### 3.3. Vehicle Communication Scenario

The vehicular scenario is shown in Figure 1, where there is a traffic jam and where an emergency vehicle needs to reach its destination through this road. Our scenario is divided into two stages. Stage 1 is where the emergency vehicle will send an emergency message to the nearest vehicle asking to clear the path. Consequently, at Stage 2, this vehicle will relay the emergency vehicle message to the nearest vehicles in the road. Next, all the vehicles will relay this message through multi-hop communication. When receiving this message, all the vehicles in the road will divert from their current path for the emergency vehicle and follow another path. Any pair of vehicles can directly communicate with each other if and only if this pair is within the radio range. Thus, the proposed model intends to find the nearest vehicle to the emergency vehicle to send an emergency message and, consequently, this message will be relayed through all the vehicles in the traffic. The proposed model adopts two communication schemes: (i) the first one is the communication between emergency vehicles and the nearest vehicle, and (ii) the second is the communication between the established vehicle (V) and all vehicles on the road, which is V2V communication. For road safety, the distance between all vehicles in the road must follow the 2 s rule, which means that the distance for V2V communication must be equal to 2 times the vehicles’ velocity. The uplink communication between any V2V pair is assumed to be via frequency-flat block-fading Rayleigh channels [36].

#### 3.3.1. First Stage

In the first stage, an algorithm is introduced to determine the nearest vehicle to the emergency vehicle that it can communicate with; this is illustrated in Algorithm 1. The nearest vehicle is determined based on different environmental conditions such as transmission power, path loss, interference, and channel noise. The interference between the required main link and the interference links are assumed to be the same for all main links. Therefore, the signal-to-interference-plus-noise (*SINR_EVN_*) and the outage probability (*p_outEVN_*) between the emergency vehicle and the nearest vehicle is given by:

(5) $$SINR_{EVN}=\frac{P_{E}{\left|h_{EVN}\right|}^{2}\;PLO\;d_{EVN}{}^{-\alpha }\;}{P_{D}{\left|h_{DVN}\right|}^{2}\;PL_{DVN}+P_{C}{\left|h_{CVN}\right|}^{2}\;PL_{CVN}+P_{vi}{\left|h_{VVN}\right|}^{2}\;PL_{VVN}+N}$$

(6) $$p_{outEVN}=1-e^{-\left(\frac{SINR_{th}\left(P_{D}{\left|h_{DVN}\right|}^{2}\;PL_{DVN}+P_{C}{\left|h_{CVN}\right|}^{2}\;PL_{CVN}+P_{vi}{\left|h_{VVN}\right|}^{2}\;PL_{VVN}+N\right)}{P_{E}{\left|h_{EVN}\right|}^{2}\;\;PLO\;d_{EVN}{}^{-\alpha }\;}\right)}$$

where *P_E_*, *h_EVN_*, *PL_O_*, and $d_{EVN}{}^{-\alpha }$ are the emergency vehicle’s transmission power, the fading channel coefficient of the vehicle, the path loss constant, and distance between the emergency vehicle and the nearest vehicle, respectively. *P_D_*, *h_DVN_,* and *PL_DVN_* are the interference device’s transmission power and fading channel coefficient, and the path loss between any transmitting device and the nearest vehicle, respectively. *P_C_*, *h_CVN_,* and *PL_CVN_* are the interference cellular user’s transmission power and fading channel coefficient, and the path loss between the transmitted CUE and the nearest vehicle, respectively. *P_vi_*, *h_VVN_,* and *PL_VVN_* are the interference vehicle’s transmission power and fading channel coefficient, and the path loss between any transmitting vehicle and the nearest vehicle, respectively. Thus, *d_EVNmax_* is constrained by the outage probability system target, so it can be deduced from Equation (6) as:

(7) $$\;d_{EVNmax}={\left(\frac{-SINR_{th}\left(P_{D}{\left|h_{DVN}\right|}^{2}\;PL_{DVN}+P_{C}{\left|h_{CVN}\right|}^{2}\;PL_{CVN}+P_{vi}{\left|h_{VVN}\right|}^{2}\;PL_{VVN}+N\right)}{P_{E}{\left|h_{EVN}\right|}^{2}\;PL_{O}\;ln\left(1-p_{outEVN}\right)}\right)}^{-\frac{1}{\alpha }}$$

#### 3.3.2. Second Stage

When the nearest vehicle receives the emergency message from the emergency vehicle, this vehicle will send this data to all the nearest vehicles in the traffic. In the proposed scenario, it is assumed that there are 4 lanes and each lane is occupied by vehicles such that the distance between each of them is assumed to be 2 multiplied by the vehicle velocity in m/s successively. The candidate nearest vehicle will send the emergency message to the nearest vehicles in each lane. Consequently, the receiving vehicles will relay the message through multi-hop communication. During this stage, Algorithm 2 is introduced to determine the vehicle system performance during the traffic congestion from the beginning of the transmission of the emergency message until the reception of this message by the last vehicle. Additionally, it shows the change in traffic conditions after reception of the emergency message by all vehicles. Thus, the outage probability of a traffic jam due to the vehicles in each lane ($p_{outLi}$) is given by:

(8) $$p_{outLi}=pout_{E2VN\to VNV1}+\overset{K}{\underset{n=1}{\prod }}pout_{liVnVn+1}-pout_{E2VN\to VNV1}*\overset{K}{\underset{n=1}{\prod }}pout_{liVnVn+1}$$

where $pout_{E2VN\to VNV1}$ is the outage probability of the emergency vehicle and the nearest vehicle, and of the nearest vehicle and the first vehicle with which it communicates in the traffic. *p_outlivnvn+_*_1_ is the outage probability of any two successive vehicles on the road. *i* is the lane number, where *i* ∈ {1, …, 4}, and *n* represents the number of vehicles in each lane in the road, where *n* ∈ {1, …, *K*}. Thus, $pout_{E2VN\to VNV1}$ and $\overset{K}{\underset{n=1}{\prod }}pout_{LiVnVn+1}$ can be expressed as:

(9) $$pout_{EVN\to VNV1}=1-\left(e^{-\left(\begin{array}{c} \frac{SINR_{th}\left(P_{D}{\left|h_{DVN}\right|}^{2}\;PL_{DVN}+P_{C}{\left|h_{CVN}\right|}^{2}\;PL_{CVN}+P_{vi}{\left|h_{VVN}\right|}^{2}\;PL_{VVN}+N\right)}{P_{v}{\left|h_{EVN}\right|}^{2}\;PL_{O}\;d_{EVN}{}^{-\alpha }}\\ +\frac{SINR_{th}\left(P_{D}{\left|h_{DV1}\right|}^{2}\;PL_{DV1}+P_{C}{\left|h_{CV1}\right|}^{2}\;PL_{CV1}+P_{vi}{\left|h_{VV1}\right|}^{2}\;PL_{VV1}+N\right)}{P_{v}{\left|h_{VNV1}\right|}^{2}\;PL_{O}\;d_{VNV1}{}^{-\alpha }} \end{array}\right)}\right)$$

(10) $$\overset{K}{\underset{n=1}{\prod }}pout_{liVnVn+1}=1-\overset{K}{\underset{n=1}{\prod }}\left(1-e^{-\left(\frac{SINR_{th}\left(P_{D}{\left|h_{DVn+1}\right|}^{2}\;PL_{DVn+1}+P_{C}{\left|h_{CVn+1}\right|}^{2}\;PL_{CVn+1}+P_{vi}{\left|h_{VVn+1}\right|}^{2}\;PL_{VVn+1}+N\right)}{P_{v}{\left|h_{VnVn+1}\right|}^{2}\;PL_{O}\;d_{VnVn+1}{}^{-\alpha }}\right)}\right)$$

Let:

1. $I_{1}=P_{D}{\left|h_{DVN}\right|}^{2}\;PL_{DVN}+P_{C}{\left|h_{CVN}\right|}^{2}\;PL_{CVN}+P_{vi}{\left|h_{VVN}\right|}^{2}\;PL_{VVN}$,
2. $I_{2}=P_{D}{\left|h_{DV1}\right|}^{2}\;PL_{DV1}+P_{C}{\left|h_{CV1}\right|}^{2}\;PL_{CV1}+P_{vi}{\left|h_{VV1}\right|}^{2}\;PL_{VV1}$, and
3. $I_{3}=P_{D}{\left|h_{DVn+1}\right|}^{2}\;PL_{DVn+1}+P_{C}{\left|h_{CVn+1}\right|}^{2}\;PL_{CVn+1}+P_{vi}{\left|h_{VVn+1}\right|}^{2}\;PL_{VVn+1}$

To ensure the model system’s performance with the road safety requirements, the communication among all the vehicles on the road should be well investigated to maximize the distance between any two vehicles on the road, and also to find the maximum velocity of each vehicle based on the channel and road conditions. Thus, for simplicity, the outage probability of the data sent from the emergency vehicle to the nearest vehicle to all the vehicles in each lane in the road can be expressed as:

(11) $$p_{outLi}=1-e^{-\left(\frac{SINR_{th}\left(I1+N\right)}{P_{v}{\left|h_{E2VN}\right|}^{2}\;PL_{O}\;d_{E2VN}{}^{-\alpha }}+\frac{SINR_{th}\left(I2+N\right)}{P_{v}{\left|h_{VNV1}\right|}^{2}\;PL_{O}\;d_{VNV1}{}^{-\alpha }}+\overset{K}{\underset{n=1}{\prod }}\frac{SINR_{th}\left(I3+N\right)}{P_{v}{\left|h_{VnVn+1}\right|}^{2}\;PL_{O}\;d_{VnVn+1}{}^{-\alpha }}\right)}$$

For road safety, the distance between any two vehicles must be greater or equal to 2 ∗ *v*; thus Equation (11) can be written as:

(12) $$p_{outli}=1-e^{-\left(\frac{SINR_{th}\left(I1+N\right)}{P_{v}{\left|h_{E2VN}\right|}^{2}\;PL_{O}\;d_{E2VN}{}^{-\alpha }}+\frac{SINR_{th}\left(I2+N\right)}{P_{v}{\left|h_{VNV1}\right|}^{2}\;PL_{O}\;d_{VNV1}{}^{-\alpha }}+\overset{K}{\underset{n=1}{\prod }}\frac{SINR_{th}\left(I3+N\right)}{P_{v}{\left|h_{VnVn+1}\right|}^{2}\;PL_{O}\;{\left(2v\right)}^{-\alpha }}\right)}$$

where *n* is the total number of vehicles on the road, which can be defined as:

(13) $$n=\frac{D}{2*v+\left(2*VL\right)}$$

where *D* and *VL* are the road length and vehicle length, respectively.

Based on Equations (12) and (13), the maximum vehicle velocity can be given as:

(14) $$v=0.5*{\left(\left(-\ln\left(1-p_{outli}\right)-\left(\frac{\;SINR_{th}\left(I_{1}+N\right)d_{E2V}{}^{\alpha }}{P_{v}{\left|h_{E2V}\right|}^{2}\;PL_{O}}+\frac{SINR_{th}\left(I2+N\right)d_{VNV1}{}^{\alpha }}{P_{v}{\left|h_{VNV1}\right|}^{2}\;PL_{O}\;}\right)\right)*\frac{\;P_{v}{\left|h_{V2V1}\right|}^{2}\;PL_{O}\;}{D*SINR_{th}*\left(I_{2}+N\right)}\right)}^{\frac{1}{\alpha -1}}$$

For the optimization problem specified in Equation (1), the first-order optimality conditions can now be investigated. The Lagrangian of the optimization problem can be calculated as:

(15) $$L\left(p_{outli},P_{v},\lambda ,\mu \right)=f\left(p_{outli},\;P_{v}\right)+\lambda \left(p_{outmax}-p_{outli}\right)+\mu \left(P_{vmax}-P_{v}\right).$$

Based on Equation (11), the distance between any two successive vehicles on the road traffic can be deduced as:

(16) $$d_{VnVn+1}={\left[\frac{P_{v}{\left|h_{VnVn+1}\right|}^{2}\;PL_{O}}{SINR_{th}\left(I3+N\right)}\left(-ln\left(1-p_{outli}\right)-\frac{SINR_{th}\left(I1+N\right)}{P_{v}{\left|h_{E2VN}\right|}^{2}\;PL_{O}\;d_{E2VN}{}^{-\alpha }}-\frac{SINR_{th}\left(I2+N\right)}{P_{v}{\left|h_{VNV1}\right|}^{2}\;PL_{O}\;d_{VNV1}{}^{-\alpha }}\right)\right]}^{-\frac{1}{\alpha }}$$

where *λ* and *μ* are non-negative Lagrangian multipliers for C1 and C2. By taking the derivative of Equation (16) concerning *p_outli_* and *P_v_*, respectively, the optimal solution to Equation (1) can be found as:

(17) $$\begin{array}{c} \frac{\partial L\left(p_{outli},P_{v},\lambda ,\mu \right)}{\partial p_{outli}}=0\\ \frac{\partial d_{VV}}{\partial p_{outli}}-\lambda =0\\ \;\;\;\;\lambda =-\frac{1}{\alpha }*{\left[\frac{P_{v}{\left|h_{VnVn+1}\right|}^{2}\;PL_{O}}{SINR_{th}\left(I3+N\right)}\left(-ln\left(1-p_{outli}\right)-\frac{SINR_{th}\left(I1+N\right)}{P_{v}{\left|h_{E2VN}\right|}^{2}\;PL_{O}\;d_{E2VN}{}^{-\alpha }}-\frac{SINR_{th}\left(I2+N\right)}{P_{v}{\left|h_{VNV1}\right|}^{2}\;PL_{O}\;d_{VNV1}{}^{-\alpha }}\right)\right]}^{-\frac{1}{\alpha }-1}*\left(\frac{1}{\left(1-p_{outli}\right)}\right) \end{array}$$

(18) $$\begin{array}{c} \frac{\partial L\left(p_{outli},P_{v},\lambda ,\mu \right)}{\partial P_{v}}=0\\ \frac{\partial d_{VV}}{\partial P_{v}}-\mu =0\\ \begin{array}{c} \mu =-\frac{1}{\alpha }*{\left[\frac{P_{v}{\left|h_{VnVn+1}\right|}^{2}\;PL_{O}}{SINR_{th}\left(I3+N\right)}\left(-ln\left(1-p_{outli}\right)-\frac{SINR_{th}\left(I1+N\right)}{P_{v}{\left|h_{E2VN}\right|}^{2}\;PL_{O}\;d_{E2VN}{}^{-\alpha }}-\frac{SINR_{th}\left(I2+N\right)}{P_{v}{\left|h_{VNV1}\right|}^{2}\;PL_{O}\;d_{VNV1}{}^{-\alpha }}\right)\right]}^{-\frac{1}{\alpha }-1}*\\ \left(\frac{{\left|h_{VnVn+1}\right|}^{2}\;PL_{O}}{SINR_{th}\left(I3+N\right)}\left(-ln\left(1-p_{outli}\right)-\frac{SINR_{th}\left(I1+N\right)}{P_{v}{\left|h_{E2VN}\right|}^{2}\;PL_{O}\;d_{E2VN}{}^{-\alpha }}-\frac{SINR_{th}\left(I2+N\right)}{P_{v}{\left|h_{VNV1}\right|}^{2}\;PL_{O}\;d_{VNV1}{}^{-\alpha }}\right)\right)+\\ \frac{P_{v}{\left|h_{VnVn+1}\right|}^{2}\;PL_{O}}{SINR_{th}\left(I3+N\right)}\left(\frac{SINR_{th}\left(I1+N\right)}{P_{v}{}^{2}{\left|h_{E2VN}\right|}^{2}\;PL_{O}\;d_{E2VN}{}^{-\alpha }}+\frac{SINR_{th}\left(I2+N\right)}{P_{v}{}^{2}{\left|h_{VNV1}\right|}^{2}\;PL_{O}\;d_{VNV1}{}^{-\alpha }}\right) \end{array} \end{array}$$

Additionally, for the value of *p_outli_* and *P_v_*, by taking the derivative of (14) concerning *λ* and *μ*, respectively, it can be found that:

(19) $$\frac{\partial L\left(p_{outli},P_{v},\lambda ,\mu \right)}{\partial \lambda }=0$$

(20) $$\frac{\partial L\left(p_{outli},P_{v},\lambda ,\mu \right)}{\partial \mu }=0$$

Next, from the first-order optimality conditions, the following two propositions can be derived. 

Proposition 1: To achieve the maximum safety distance after receiving the emergency message with the best required QoS between any two vehicles on the road, the outage probability for each lane (*p_outli_*) should be equal to the maximum required the outage probability (*p_outmax_)*, which is given by:

*p_outli_* = *p_outmax_* (21)

Proposition 2: To achieve the maximum safety distance after receiving the emergency message with the best required QoS, the transmission power of each vehicle (*P_v_)* should be equal to the maximum vehicle transmission (*P_vmax_)*, which is given by:

*P_v_* = *P_vmax_* (22)

The two expressions obtained from Equations (21) and (22) play a significant role in enhancing the system’s performance, determining the required safety distance between vehicles and the maximum required velocity. In addition, determining the maximum required safety distance between vehicles will guide the driver as to which velocity he/she should drive at and which path he/she should follow. Additionally, controlling the vehicle transmission power helps decrease the overall system power consumption and the cost as well; it also decreases the interference among other existing transmission sources.
**Algorithm 1**: Finding the nearest vehicle with which to communicate**Input**: Number of vehicles in the transmission area (*V*), emergency vehicle position, emergency vheicle transmisson power (*P_E_*), channel fade coefficient (*h_EVN_*), different interfence devices, interference transmission power (*P_D_*, *P_C_*, *P_vi_*), interference channel fade coefficient (*h_DVN_*, *h_CVN_*, *h_VVN_*), channel noise (*N*), the required outage probability (*p_outEVN_*), and the path loss exponent (*α*) **Output**: Finding the nearest vehicle with which to communicate 1: Establish the required *p_outEVN_* 2: **for** different values of *P_E_*, *P_D_*, *P_C_*, *P_v_*, *h_EVN_*, *h_DVN_*, *h_CVN_*, *h_VVN_*, *α*
**do** 3: Determine the nearest receiving vehicle using $\;d_{EVNmax}={\left(\frac{-SINR_{th}\left(P_{D}{\left|h_{DVN}\right|}^{2}\;PL_{DVN}+P_{C}{\left|h_{CVN}\right|}^{2}\;PL_{CVN}+P_{vi}{\left|h_{VVN}\right|}^{2}\;PL_{VVN}+N\right)}{P_{E}{\left|h_{EVN}\right|}^{2}\;PL_{O}\;ln\left(1-p_{outEVN}\right)}\right)}^{-\frac{1}{\alpha }}$ 4: Send emergency message to the nearest receiver directly 5: **end for**

**Algorithm 2**: System performance evaluation**Input**: Number of lanes (*L*), initial number of vehicles (*n_i_*) in each lane lane, emergency vehicle position, emergency vehicle transmisson power (*P_E_*), vehicle transmisson power (*P_v_*), channel fade coefficient (*h_EVN_*, *h_VNV1_*), different interfence devices, interference transmission power (*P_D_*, *P_C_*, *P_vi_*), interference channel fade coefficient (*h_DV1_*, *h_CV1_*, *h_VV1_*), channel noise (*N*), path loss exponent (*α*), and the initial vehicle velocity (*v_i_*) **Output**: PDR, E2E, *v*, number of vehicles in each lane (*n*), *p_outLi_*, *P_v_* 1: Establish the required $pout_{Li}$ 2: Establish *L*, *n_i_*, *v_i_* 3: **for** different values of *P_E_*, *P_D_*, *P_C_*, *P_v_*, *h_EVN_*, *h_DVN_*, *h_CVN_*, *h_VVN_*, *α*, *v*, *N*
**do** 4: Calculate the velocity of the vehicles in each lane after receiving the emergency message $v=0.5*{\left(\left(-\ln\left(1-p_{outli}\right)-\left(\frac{\;SINR_{th}\left(I_{1}+N\right)d_{E2V}{}^{\alpha }}{P_{v}{\left|h_{E2V}\right|}^{2}\;PL_{O}}+\frac{SINR_{th}\left(I2+N\right)d_{VNV1}{}^{\alpha }}{P_{v}{\left|h_{VNV1}\right|}^{2}\;PL_{O}\;}\right)\right)*\frac{\;P_{v}{\left|h_{V2V1}\right|}^{2}\;PL_{O}\;}{D*SINR_{th}*\left(I_{2}+N\right)}\right)}^{\frac{1}{\alpha -1}}$; 5: Calculate required number of vehicles in each lane $n=\frac{D}{2*v+\left(2*VL\right)}$
6: Construct the Lagrange function    $L\left(p_{outli},P_{v},\lambda ,\mu \right)=f\left(p_{outli},\;P_{v}\right)+\lambda \left(p_{outmax}-p_{outli}\right)+\mu \left(P_{vmax}-P_{v}\right)$ 7: Calculate the partial derivatives $\frac{\partial L\left(p_{outli},P_{v},\lambda ,\mu \right)}{\partial \lambda }$,$\;\frac{\partial L\left(p_{outli},P_{v},\lambda ,\mu \right)}{\partial \mu },\;\;\frac{\partial L\left(p_{outli},P_{v},\lambda ,\mu \right)}{\partial p_{outli}}\;$, and $\frac{\partial L\left(p_{outli},P_{v},\lambda ,\mu \right)}{\partial P_{v}}$ 8: Let $\frac{\partial L\left(p_{outli},P_{v},\lambda ,\mu \right)}{\partial \lambda }=0$, $\frac{\partial L\left(p_{outli},P_{v},\lambda ,\mu \right)}{\partial \mu }=0,$ $\frac{\partial L\left(p_{outli},P_{v},\lambda ,\mu \right)}{\partial p_{outli}}=0$, $\frac{\partial L\left(p_{outli},P_{v},\lambda ,\mu \right)}{\partial P_{v}}=0$ 9: Determine ***𝛌***, μ, *p_outLi_*, *P_v_* 10: Calculate PDR              $PDR=\frac{\#received\_packets}{\#sent\_packets}$ 11: Calculate E2E       $E2E=\frac{\sum_{\mathrm{received}\;\mathrm{packets}}\mathrm{time}\;\mathrm{spent}\;\mathrm{to}\;\mathrm{deliver}\;\mathrm{packets}}{\#\mathrm{received}\_\mathrm{packets}}$ 12: end for

Two metrics have been considered to evaluate the system performance of the proposed model and compare it with the model proposed in [19], which are:

4 Packet delivery ratio (*PDR*): This is the ratio of the successful number of packets received by the destinations to the total number of the packets sent by the source. The system is considered reliable and efficient when the value of PDR increases. Thus, PDR can be given as [37]:

(23) $$PDR=\frac{\#received\_packets}{\#sent\_packets}$$

5 Average end-to-end delay (*E2E*): The average end-to-end delay can be defined as the total time needed to successfully receive the transmitted packets over a network. The average end-to-end delay can be measured as the sum of the amount of time spent from sending a packet until it reaches its destination over the total number of packets received by the destination [37]:

(24) $$E2E=\frac{\sum_{\mathrm{received}\;\mathrm{packets}}\mathrm{time}\;\mathrm{spent}\;\mathrm{to}\;\mathrm{deliver}\;\mathrm{packets}}{\#\mathrm{received}\_\mathrm{packets}}$$

## 4. Numerical Results and Discussion

### 4.1. Introduction

In this section, the performance of the proposed traffic management technique was evaluated through two different software simulation packages: MATLAB and NS-2.

### 4.2. Software Tools

The MATLAB software package was used to implement the mathematical model for finding the maximum distance between the emergency vehicle and the first receiving vehicle under different channel conditions such as (i) transmission interference power, (ii) distance to interference, and (iii) signal-to-interference-plus-noise (*SINR*). Meanwhile, the NS-2 software package is considered as very powerful simulation software, so it was used for evaluating the system performance in terms of (i) packet delivery ratio and (ii) average end-to-end delay.

### 4.3. Investigated Case Parameters

For avoiding collisions and reducing the rate of accidents, the 2 s rule was followed all over the road [30]. Following this rule, each driver must keep a sufficient distance between his/her vehicle and any other vehicle in front for his/her safety.

NS-2 was used to build the road traffic with its conditions, besides the mobility files generated. Additionally, to evaluate the system performance, an AWK script was implemented to extract the required measured metrics from trace files generated during the simulation. Moreover, in the suggested proposed traffic management technique, all vehicles were running at a fixed speed which was 60 km/h as long as they had not received the emergency message. When the emergency message was received, the vehicles’ velocity changed. Based on road traffic information, the lane width in the road was 3.5 m and the distance between two consecutive vehicles at any stage followed the 2 s rule [30]. The network parameters used for simulation are listed in Table 1 [19,38,39,40,41].

### 4.4. Results Discussion

**Figure 2:**
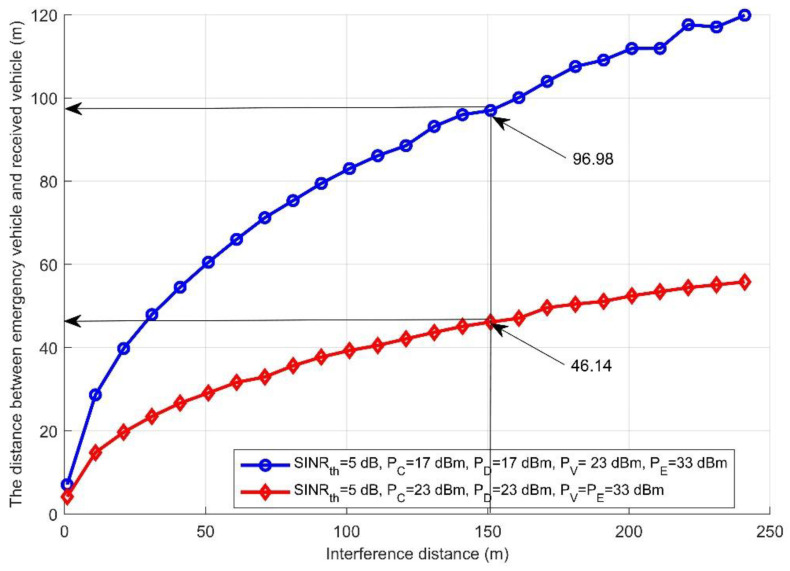
The distance between the emergency vehicle and the receiving vehicle versus interference distance.

Based on Algorithm 1, Figure 2 depicts the distance of the nearest vehicle to the emergency vehicle that it can communicate with versus the interference distance under different conditions such as different interference and *SINR.* Assuming that there are two scenarios, in the first one, *SINR* is 5 dB, and the interference transmission power of the CUE, D2D links, and V2V link is 17 dBm, 17 dBm, and 23 dBm, respectively; this case is considered low interference transmission power. However, in the second, the interference transmission power of the CUE, D2D links, and V2V links is 23 dBm, 23 dBm, and 33 dBm, respectively, which is considered high interference transmission power. As can be observed, when both scenarios were considered, the distance to reach the nearest vehicle dramatically increased when the interference distance increased. When the two scenarios were compared, it was found that using low interference transmission power allowed the emergency message to be sent and successfully received with the longest possible distance e.g., when the interference distance was 151 m, the nearest receiving vehicle can be at a distance equal to 96.98 m. However, when using high interference transmission power for the same interference distance, 151 m, the nearest receiving vehicle can be at distance equal to 46.14 m. It can be concluded from this figure that the interference transmission power can prevent the sent message reaching the nearest vehicles and thus prevent the emergency vehicle reaching its destination with minimum travel time.

**Figure 3:**
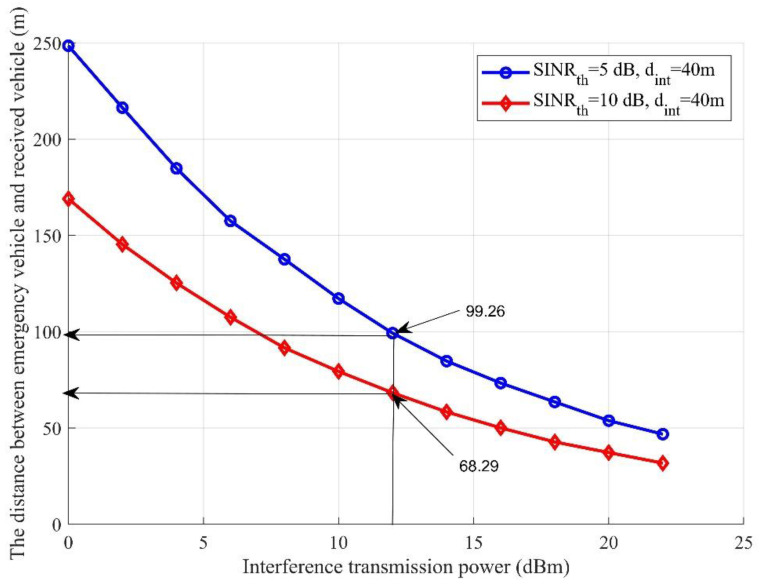
The distance between the emergency vehicle and the receiving vehicle versus interference transmission power.

The distance of the nearest vehicle to the emergency vehicle with which it can communicate is illustrated, again based on Algorithm 1, in Figure 3 with different values of interference transmission. Figure 3 demonstrates that when the transmission power of the interfering devices increased, the emergency message could not be correctly received at very long distances. This means that when the interference transmission power increased the vehicles in the nearest area could not receive the correct message due to the high interference. Two different scenarios have been examined, where the interference distance was assumed to be 40 m with two different required *SINR* (*SINR_th_*) values. It can be noticed that when *SINR_th_* was equal to 10 dB and the interference transmission power was 12 dBm, the nearest vehicle’s distance to correctly receive the message under this condition must be 68.29 m. However, when *SINR_th_* was equal to 5 dB and the interference transmission power was 12 dBm, the distance to the nearest vehicle should be 99.26 m to correctly receive the emergency message. The result obtained in Figure 3 is correlated with the corresponding performance obtained in Figure 2.

**Figure 4:**
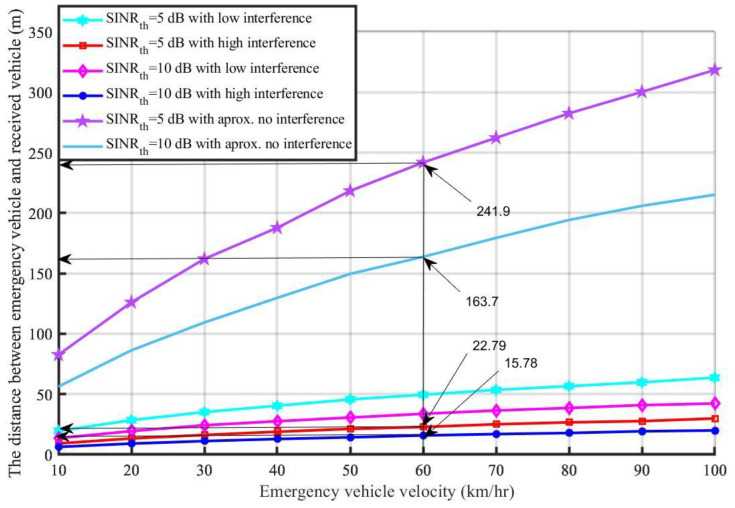
The distance between the emergency vehicle and the receiving vehicle versus the emergency vehicle’s velocity.

Based on the introduced Algorithm 1, Figure 4 shows the effect of the interference level on the distance that the emergency message travels with different emergency vehicle velocities. It should be mentioned that the distance to reach the nearest vehicle increased when the emergency vehicle’s velocity increased. Additionally, when the interference increased with high *SINR_th_*, the distance required to reach the nearest vehicles decreased; e.g., when *SINR_th_* was equal to 10 dB, the emergency vehicle’s velocity was 60 km/h, and the system faced high interference, the maximum distance the message could travel was 15.78 m. However, when *SINR_th_* was equal to 5 under the same conditions, the message could travel up to 22.79 m. On the other hand, when there was no interference, it could be noticed that the distance that the emergency message traveled became 163.7 m and 241.9 m for when *SINR_th_* equaled 10 dB and 5 dB, respectively, with the same channel and road traffic conditions. It can be concluded from Figure 4 that interference can affect the traffic safety and transmission efficiency because if the sent message cannot reach its destination effectively, this could influence the traffic safety because a lack of information and V2V communication will occur. This may lead to a traffic collision and accidents.

### 4.5. Comparative Analysis

**Figure 5:**
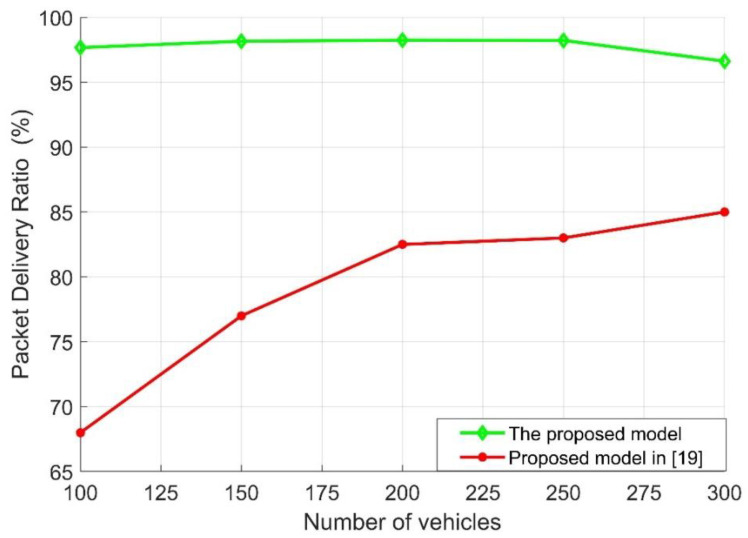
Packet delivery ratio [PDR] vs. number of vehicles.

For fair assessment, the proposed traffic management strategy was compared with the recent evaluated model presented in [19]. The model presented in [19] mainly focused on precise vehicle position to decide a suitable route. The model presented in [19] also selected the link with the highest reliability as the transmission path (choosing the path with high *SINR*) without taking the worst environmental conditions into consideration. Figure 5 demonstrates the packet delivery ratio (PDR) under different numbers of vehicles in the road compared with the multi-level dynamic link model presented in [19]. As can be seen and determined from the proposed Algorithm 2, when the number of vehicles increased, the connectivity of the network was greatly improved, and then the packet delivery ratios of both models increased. It can be also mentioned that the PDR of the proposed model partially increased compared with the other model, which gradually increased, as the main purpose of the proposed model is enhancing the system’s connectivity through V2V communication with an increase in traffic safety. Additionally, it can be seen that the PDR of the proposed model started to decrease when the number of vehicles was 300 but still outperformed the model presented in [19]. This was due to the network’s overhead and the interference caused by the sender vehicles. Furthermore, the model presented in [19] assumed that the *SINR_th_* was 20 dB, which means that the interference and the channel contention presented will not effectively affect the network performance. However, in the proposed model, the worst case scenarios were considered, namely that *SINR_th_* varied from 5 to 10, only to show how the interference and the channel contention would affect the proposed model.

**Figure 6:**
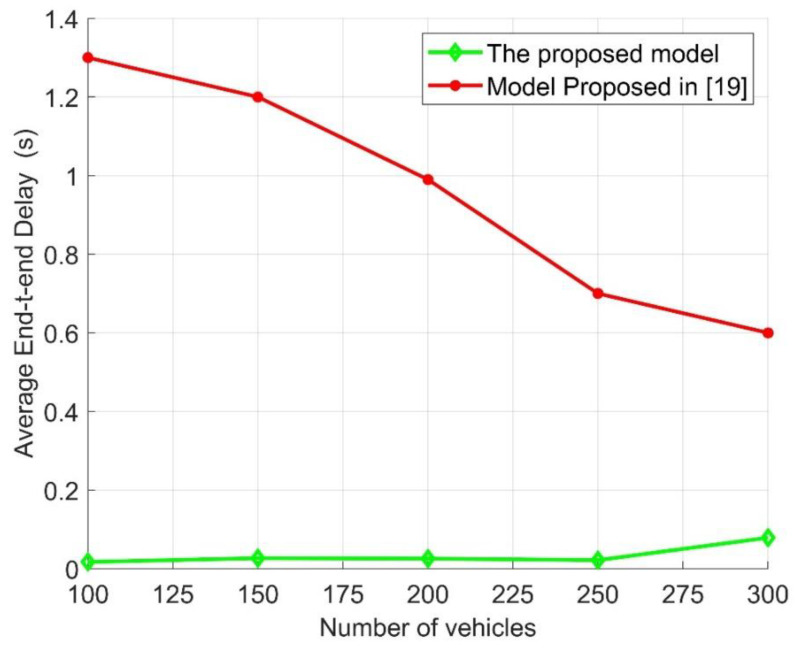
Average end-to-end delay [E2E] versus the number of vehicles.

As proven in Algorithm 2, Figure 6 illustrates the average end-to-end delay (E2E) under different numbers of vehicles with the same channel, network, and traffic conditions. By comparing the proposed model with the presented model in [19], it can be found that the proposed model had a lower average end-to-end delay, as increasing the number of the vehicles under the assumed assumption for both models increased the average end-to-end delay. It is worth mentioning that the increment in the E2E of the proposed model was partially increased compared with the model presented in [19], which gradually increased when the number of vehicles increased. The results obtained show the strength of the proposed model and show how when the number of vehicles increases, the E2E increases slightly, which is an important factor that could affect traffic safety. Additionally, it can also be seen that for the proposed model, when the number of vehicles was 300, the E2E increased gradually; this was due to the decrease in the successfully received packets and the increase in the number of retransmission packets compared with the number of other vehicles. The performance obtained was similar to the packet delivery rate, in that the network interference and channel contention slightly affected the proposed model network performance. Compared with the model presented in [19], the proposed model achieved a better PDR and E2E for different numbers of vehicles under different channel and traffic conditions. Through this performance, the proposed traffic management system showed the effectiveness of the proposed model in terms of network connectivity and traffic safety.

**Figure 7:**
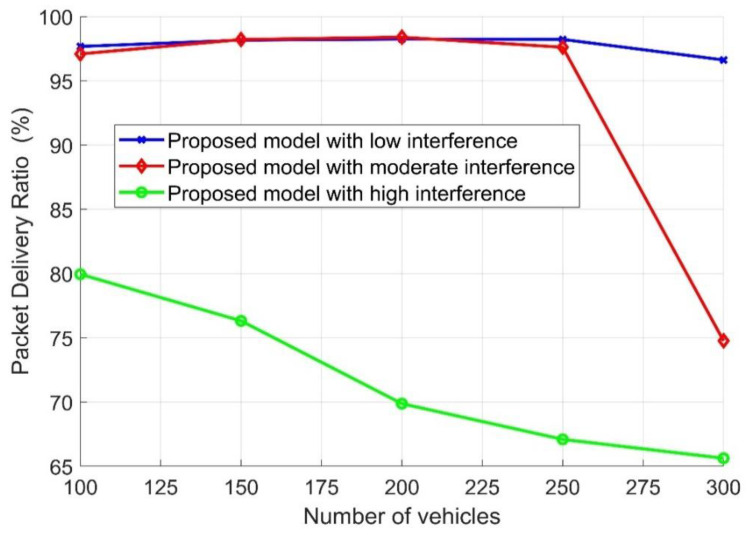
Packet delivery ratio versus the number of vehicles.

The packet delivery ratio (PDR) with different numbers of vehicles in the road for the proposed traffic management strategy is illustrated in Figure 7, once again based on the introduced Algorithm 2 and evaluated once again for different interference levels, assuming 17 links, 37 links, and 75 links for low, moderate, and high interference, respectively. As demonstrated in Figure 7, when the network faced high interference with an increase in the number of vehicles, the PDR gradually decreased due to the network’s overhead. Furthermore, it can be seen that when the network experienced low and moderate interference, the PDR slightly increased when the number of vehicles increased. The PDR increased when the number of vehicles increased from 100 to 250 due to the increment in the number of packets generated. Thus, when the number of vehicles was 300, the PDR decreased due to the network overhead caused by the number of transmitting and interfering nodes.

### 4.6. Interference Effect

**Figure 8:**
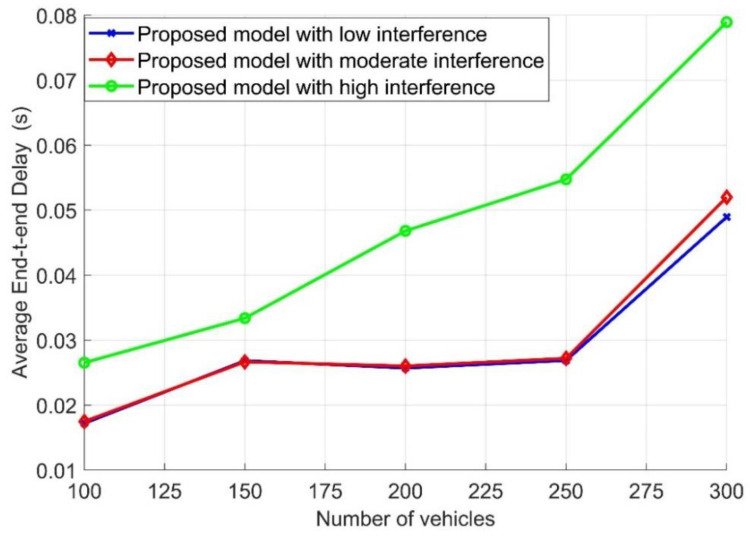
Average end-to-end delay (E2E) versus the number of vehicles.

For the same conditions as mentioned in Figure 7 and following Algorithm 2, the average end-to-end delay (E2E) with different numbers of vehicles in the road is examined in Figure 8 for different interference levels. As illustrated in Figure 8, the E2E of the proposed model decreased when the network faced low and moderate interference compared with high interference. Additionally, for low and moderate interference, the E2E dramatically increased when the number of vehicles was 300 due to the increase in retransmission time caused by the network’s overhead and interference. Furthermore, with high interference, the E2E fluctuated based on the number of received packets, which was strongly influenced by the interference. This result is correlated with the result obtained in Figure 7. It can also be noticed that when the number of vehicles was 300, the E2E with high interference was higher than that with moderate and low interference. In the case of high interference, the number of received packets decreased due to packet loss and the number of retransmission packets. However, in the case of low and moderate interference, the number of received packets increases and then the E2E increased.

### 4.7. Emergency Vehicle Travel Time

**Figure 9:**
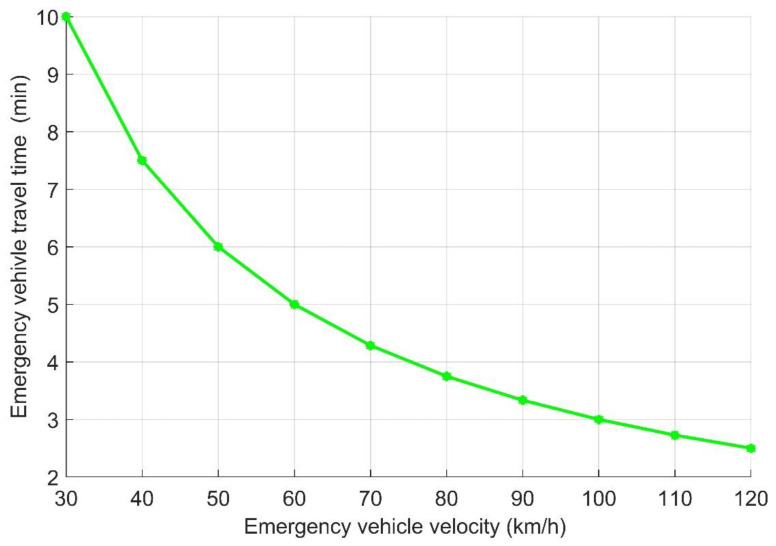
Emergency vehicle travel time versus emergency vehicle velocity.

Based on Algorithm 2, the emergency vehicle travel time was evaluated with different emergency vehicle velocities. As can be observed from Figure 9, based on the proposed model, if the distance between the emergency vehicle and its destination was 5000 m. The time taken to reach its destination after applying the proposed model to the traffic road varied based on the emergency vehicle’s velocity; e.g., if the emergency vehicle’s velocity was 100 km/h, which is closer to real life, and the other vehicles in the road clear the path based on the proposed idea, the emergency vehicle would take only 3 min to reach its destination, which is a distance of 5000 m. This result shows how the proposed model could save people’s lives and property, as if the emergency follows the normal road to reach its destination, it may take time more than the result obtained by approximately 25% or more. A delay of 25% or more to reach its destination could make others lose their lives. That is why the proposed model is considered as one of the solutions to solve the problem of congestion, at least for emergency vehicles.

### 4.8. Proposed Enhancement Adaptive Algorithm

Furthermore, an adaptive algorithm was proposed, based on the introduced Algorithm 2. This adaptive algorithm can be obtained from the derived Equations (13) and (14) stated in Section 3. These derived equations show how the vehicle’s velocity and distance will change in each lane based on the sent information and the environmental conditions. It also shows the required safety distance between any two successive vehicles; this distance is calculated based on the well-known worldwide 2 s rule. Table 2 shows how the vehicle performance varied before and after receiving the emergency message. It can be noticed that when the vehicle velocity was 60 km/h and the road length was 1000 m, based on the 2 s rule, each lane before receiving the emergency message had approximately 26 vehicles and the safety distance between vehicles was approximately 33.33 m. However, when it had received the emergency message, the vehicle’s velocity reduced to 19.8838 km/h to allow other vehicles to follow its lane; each lane had approximately 65 vehicles with a safe distance between each vehicle equal to 10.2 m.

The performance evaluation presented in Figure 2, Figure 3, Figure 4, Figure 5, Figure 6, Figure 7, Figure 8 and Figure 9 shows how the proposed model presented an effective model for V2V communication to increase the system’s reliability and the effectiveness of the connectivity and, at the same time, decrease the travel time for the emergency vehicle to its destination. Furthermore, it was noted that many factors are affecting the V2V connection such as path loss, channel condition, vehicle mobility, density, vehicle velocity, and interference. It was also shown that one of the suitable and adequate solutions is the proposed model, by finding the nearest vehicle to the emergency vehicle with which to communicate to relay the information to the vehicles in the road. Based on the emergency message sent, all the vehicles on the road will adapt their velocity and follow another path to clear the road for the emergency vehicle. The proposed model helps decrease the emergency vehicle travel time; at the same time, it helps increase system reliability and the V2V connectivity. To enhance the system’s performance and achieve the minimum travel time for the emergency vehicle as clarified in this paper, the nearest vehicle to the emergency vehicle with which it can communicate should be determined based on the system conditions and requirements such as the outage probability (*P_out_*), the transmission power *P_v_* and channel quality in terms of *α,* the vehicles’ mobility, the vehicles’ density, the vehicles’ velocity, and interference. The findings of this work can assist in deciding with which vehicle the connection should be established to relay the emergency information to the other vehicles on the road. Additionally, based on the information sent, some of the vehicles will change their velocity and the rest of the vehicles will decrease their velocity and follow another path. By clearing the path for the emergency vehicle, this will help it to arrive at its destination with the minimum possible travel time.

## 5. Conclusions

A new traffic management model through V2V communications was proposed to control traffic jams and allow the emergency vehicles to reach their destination safely with the minimum travel time. The nearest vehicle found based on the proposed model helped to reduce the emergency vehicle’s travel time by relaying the emergency message to the other vehicles to clear the road. Additionally, based on the received information, the vehicles in the road adapted their velocity and the maximum distance between any two vehicles to avoid collisions and accidents. Based on the Lagrange optimization technique, the maximum distance between every two vehicles and the maximum velocity for each vehicle was determined under different channel and traffic conditions. Based on the system performance evaluation under the severe channel and traffic conditions, it has been shown that the proposed model can exhibit the best performance under certain environmental conditions compared with other proposed models. The maximum distance between the emergency vehicle and the nearest vehicle can be identified based on the communication environment, such as how many devices can interfere with the E2V communication and the velocity of the vehicle. The results presented in this study can be used to form an adaptive model to control the vehicles’ mobility and direction when receiving an emergency message to achieve the minimum required emergency vehicle travel time with the best V2V transmission efficiency.

## Figures and Tables

**Table 1:** Simulation parameters.

*Parameters*	Value
No	−144 dBm
B	10 MHz
P_vmax_	33 dBm
P_D_	17 or 23 dBm
P_C_	17 or 23 dBm
Packet size	512 bytes
fc	5.9 GHz
Simulation time	1000 s
Number of vehicles	100~300
Speed	60 km/h
Traffic agent	CBR
Queue	PriQueue with a size of 50 packets
Propagation mode	Two-way model
Antenna	Omni-directional with a height of 1 m
Routing Protocol	DSRC
Number of seeds	3
Noise	AWGN
PL_V2V_	127 + 30 log_10_(*d_VV_*)
PL_CBS_	128.1 + 30 log_10_(*d_CBS_*)
PL_D2D_	148 + 30 log_10_(*d_DD_*)

**Table 2:** Traffic and vehicle performance before and after receiving the emergency message.

Vehicle Performance Before Receiving an Emergency Message			Vehicle Performance after Receiving an Emergency Message		
Vehicle Velocity (km/h)	Number of Vehicles per 1 km	Safe Distance between Vehicles (m)	Vehicle Velocity (km/h)	Number of Vehicles per 1 km	Safe Distance between Vehicles (m)
30	46.1538	16.66667	10.9821	90.0807	6.1012
40	36.7347	22.22222	13.4790	80.0749	7.4883
50	30.5085	27.777778	15.6964	72.8851	8.7202
60	26.0870	33.333333	19.8838	65.7786	10.2025
70	22.7848	38.888889	22.3220	62.3188	11.0465
80	20.2247	44.444444	24.5729	57.4675	12.4011
90	18.1818	50	25.9517	53.6146	13.6516
100	16.5138	55.555556	28.4307	51.4997	14.4176
110	15.1261	61.111111	29.6037	48.0889	15.7948
120	13.9535	66.6666667	29.9143	46.6277	16.4465

## Data Availability

Not applicable.

## Symbols

QoS	Quality of service
*C1, C2*	Lagrangian optimization constraints
*d_VnVn+1_*	Distance between two successive vehicles in the road
*P_D_*	The transmission power of any interference device
*P_Dmax_*	The maximum transmission power of any transmission device
*P_C_*	The transmission power of any interference cellular user’s equipment
*P_Cmax_*	The maximum transmission power of any cellular user’s equipment
*P_v_*	The transmission power of any vehicle
*P_vi_*	The transmission power of any interference vehicle
*P_vmax_*	The maximum transmission power of any vehicle
$h_{VnVn+1}$	Fading channel coefficient between any two successive vehicles
$h_{DVn+1}$	Fading channel coefficient between the interfering device and the receiving vehicle
$h_{CVn+1}$	Fading channel coefficient between the interfering CUE and the receiving vehicle
$h_{VVn+1}$	Fading channel coefficient between the interfering vehicle and the receiving vehicle
$PL_{DVn+1}$	Path loss between any interfering device and the receiving vehicle
$PL_{CVn+1}$	Path loss between any interfering CUE and receiving vehicle
$PL_{VVn+1}$	Path loss between any interfering vehicle and receiving vehicle
$PL_{VnVn+1}$	Path loss between any two V2V links
*PDR*	Packet delivery ratio
*E2E*	Average end-to-end delay
*p_out_*	System outage probability
*p_outmax_*	Maximum system outage probability
SINR	Signal-to-interference-plus-noise ratio
$SINR_{th}$	Signal-to-interference-plus-noise ratio threshold
*SINR_EVN_*	Signal-to-interference-plus-noise between the emergency vehicle and the nearest vehicle
*p_outEVN_*	Outage probability of the emergency vehicle and the nearest vehicle
*d_EVN_*	Distance between the emergency vehicle and the nearest vehicle
*d_EVmax_*	Maximum distance between the emergency vehicle and the nearest vehicle
h	Fading channel coefficient
N	Noise power spectral density
*P_S_*	Source transmission power
γ	Path loss between the source and destination
$P_{E}$	Emergency vehicle’s transmission power
$h_{EVN}$	Fading channel coefficient between the emergency vehicle and the nearest vehicle
$PL_{EVN}$	Path loss between the emergency vehicle and the nearest vehicle
$h_{DVN}$	Fading channel coefficient between the interfering device and the nearest vehicle
$PL_{DVN}$	Path loss between theinterfering device and the nearest vehicle
$h_{CVN}$	Fading channel coefficient between theinterfering CUE and the nearest vehicle
$PL_{CVN}$	Path loss between the interfering CUE and the nearest vehicle
$h_{VVN}$	Fading channel coefficient between the interfering vehicle and the nearest vehicle
$PL_{VVN}$	Path loss between the interfering vehicle and the nearest vehicle
*p_outEVN_*	Outage probability of the emergency vehicle and the nearest vehicle
*p_outli_*	Outage probability of all the communication links in the lane *i*
$pout_{E2VN\to VNV1}$	Outage probability of the emergency vehicle and the nearest vehicle, and the nearest vehicle and the first vehicle in the traffic
$pout_{liVnVn+1}$	Outage probability of all communicating V2V links in the lane *i*
$h_{VNV1}$	Fading channel coefficient between the nearest vehicle and the first receiving vehicle on the road
$PL_{VNV1}$	Path loss between the emergency vehicle and the nearest vehicle
$h_{DV1}$	Fading channel coefficient between the nearest vehicle and the first receiving vehicle on the road
$PL_{DV1}$	Path loss between the interfering device and first receiving vehicle on the road
$h_{CV1}$	Fading channel coefficient between the interfering CUE and the first receiving vehicle on the road
$PL_{CV1}$	Path loss between the interfering CUE and the first receiving vehicle on the road
$h_{VV1}$	Fading channel coefficient between the interfering vehicle and the first receiving vehicle on the road
$PL_{VV1}$	Path loss between the interfering vehicle and the first receiving vehicle on the road
$PL_{O}$	Path loss constant
I	Interference
*n*	Number of vehicles on the road
D	Road length
*v*	Vehicle velocity
*VL*	Vehicle length
λ, µ	Non-negative Lagrangian multipliers
